# Erinacine C Attenuates Alzheimer’s-like Pathology: A Study in APP/PS1 Mice and PC12 Cells

**DOI:** 10.3390/molecules31183154

**Published:** 2026-09-08

**Authors:** Li-Yu Wang, Shu-Lan Yeh, Shih-Tien Hsu, Shu-Ming Huang, Chi-Fai Chau, Cheng-Hung Chuang

**Affiliations:** 1 Department of Nutrition, Hungkuang University, Taichung 43302, Taiwan; 2 Department of Food Science and Biotechnology, National Chung Hsing University, Taichung 40227, Taiwan; 3 Department of Nutrition, Chung Shan Medical University, Taichung 40203, Taiwan; 4 Department of Obstetrics and Gynecology and Women’s Health, Taichung Veterans General Hospital, Taichung 40705, Taiwan; 5 Department of Nutrition, Nantou Hospital of Ministry of Health and Welfare, Nantou 54062, Taiwan

**Keywords:** erinacine C, *Hericium erinaceus*, Alzheimer’s disease, Akt/GSK3β signaling, neuroinflammation, apoptosis, APP/PS1 mice, tau phosphorylation

## Abstract

Alzheimer’s disease (AD) is a progressive neurodegenerative disorder characterized by amyloid-beta (Aβ) deposition and cognitive decline. Clinical evidence indicates that *Hericium erinaceus* whole fruiting body powder offers limited therapeutic efficacy, which is suggested to be constrained by whole-food matrix interference and uncertainties in central nervous system (CNS) exposure. Therefore, we hypothesized that erinacine C (EC)—a purified active component from *Hericium erinaceus* mycelia—could resolve these limitations and potentially exhibit favorable neuroprotective effects owing to its low molecular weight and lipophilicity. In this study, we investigated the neuroprotective potential and associated signaling alterations of EC using APP/PS1 transgenic mice and Aβ_25-35_-induced PC12 cells. In vivo, oral administration of EC alleviated deficits in activities of daily living, as evidenced by improvements in nesting and burrowing behaviors, along with enhanced short-term spatial memory in the Y-maze test. EC treatment was associated with a reduction in hippocampal Aβ plaque accumulation, suppression of glial activation (GFAP and IBA1), and decreased IL-6 levels in both serum and hippocampal tissues. In vitro, EC intervention counteracted Aβ_25-35_-induced cytotoxicity in PC12 cells. Analysis of signaling markers revealed that EC treatment was accompanied by a restoration of p-Akt/Akt levels and a suppression of p-GSK3β (Tyr216) activation. Consequently, EC treatment was correlated with reduced tau hyperphosphorylation, the up-regulation of the anti-apoptotic protein Bcl-2, and the down-regulation of pro-apoptotic markers including Bax and cleaved-caspase-3, thereby lowering the total apoptotic rate. Taken together, these findings suggest that EC may mitigate cognitive impairment and neurodegeneration in parallel with alterations in the Akt/GSK3β/tau/caspase-3 signaling response, thereby representing a potentially promising therapeutic candidate for the intervention of AD.

## 1. Introduction

As the global population ages, dementia has emerged as a formidable public health challenge. Currently, approximately 55 million people worldwide are living with dementia, a figure projected to escalate to 139 million by 2050. Alzheimer’s disease (AD), a highly prevalent chronic neurodegenerative disorder, accounts for nearly 60% of these cases [1,2]. AD is clinically characterized by progressive memory loss, a decline in activities of daily living (ADL) and cognitive impairment [2,3,4]. Pathologically, it is defined by the extracellular accumulation of amyloid-beta (Aβ) plaques and the intracellular formation of neurofibrillary tangles (NFTs), which lead to irreversible neuronal damage and cognitive decline.

The over-deposition of Aβ in the cerebral cortex and hippocampus is widely recognized as the primary pathogenic initiator of AD [5,6]. Specifically, the Aβ_25-35_ fragment exhibits potent physiological toxicity, directly inducing neuronal apoptosis [7,8]. Furthermore, Aβ accumulation triggers the activation of microglia and astrocytes, shifting them from a quiescent state to an active pro-inflammatory phenotype. This transition results in the excessive release of pro-inflammatory cytokines, such as Interleukin (IL)-6 [7,8], tumor necrosis factor-α (TNF-α) [9], and IL-1β [10,11]. This sustained neuroinflammatory response, intertwined with oxidative stress, creates a vicious cycle that ultimately leads to neuronal apoptosis and synaptic dysfunction, thereby resulting in progressive cognitive impairment [12,13,14].

Among the mechanisms of Aβ-induced cytotoxicity, the Akt (Protein Kinase B)/GSK3β (glycogen synthase kinase 3 beta) signaling pathway is regarded as a central regulator of neuronal fate [15,16]. Akt is a crucial upstream survival factor that participates in regulating GSK3β activity via phosphorylation at the Ser9 site, which is closely associated with maintaining tau stability and regulating apoptotic signaling [16,17]. However, under the pathological stress of Aβ, Akt activation is significantly diminished, leading to altered regulation and phosphorylation of GSK3β.

Activated GSK3β, particularly when phosphorylated at the Tyr216 site, is associated with the abnormal hyperphosphorylation of tau—contributing to NFT—as well as the modulation of Bcl-2 and Bax expression. This shifts the balance toward apoptosis in parallel with the activation of the Caspase-3 cascade [18,19].

*Hericium erinaceus*, a basidiomycete fungus, has a centuries-long history of culinary and medicinal use in East Asia. Modern research has confirmed its diverse biological activities, including antioxidant [20,21,22], anti-inflammatory [23,24,25], and neuroprotective properties [26,27,28]. Although consuming the functional whole-food matrix of *Hericium erinaceus* provides broad health benefits, its direct application for targeted AD intervention faces certain pharmacological limitations. First, the complex whole-food matrix introduces significant molecular interference and chemical variability, which can create substantial uncertainties regarding the pharmacokinetic profiles and central nervous system (CNS) exposure efficiency of its active components [29]. Second, the profiles and concentrations of secondary metabolites within the whole mushrooms fluctuate depending on the cultivation substrates and growth environments, posing challenges for the development of standardized therapeutic protocols [30]. Furthermore, while the general populace primarily consumes the mushrooms as a whole food, the neuroprotective cyathane-type diterpenoids are uniquely enriched in the mycelia but remain scarce in the fruiting bodies [31,32]. Therefore, the strategic isolation and characterization of specific functional components are highly necessary to minimize matrix interference and achieve a consistent bioactive evaluation.

Among these mycelia-derived bioactive components, erinacines represent a group of cyathane-type diterpenoids with neuroprotective potential [33,34,35]. Notably, erinacine C (EC) exhibits unique biological potential. The low molecular weight and high lipophilicity of EC are theoretically considered advantageous for potential BBB penetration [32,36,37], and this compound has been shown to stimulate the synthesis of nerve growth factor (NGF) [36]. Despite these promising traits, available research on EC remains notably limited, and the precise molecular mechanisms associated with its capacity to counteract cognitive deficits and attenuate neuroapoptosis—particularly in relation to the Akt/GSK3β axis—warrant systematic elucidation in both animal and cellular models.

Based on these premises, we hypothesized that the isolated diterpenoid EC could effectively alleviate Aβ-induced neurodegenerative cascades in parallel with alterations in the Akt/GSK3β survival axis, offering a more targeted intervention compared to non-standardized food matrix extracts. To address this, the present study evaluated the therapeutic effects of EC on cognitive impairment, cerebral Aβ deposition, and neuroinflammation using APP/PS1 transgenic mice. Concurrently, we utilized an Aβ-induced PC12 cell model to verify, at the molecular level, whether the neuroprotective actions of EC are accompanied by the modulation of the Akt/GSK3β signaling response, a reduction in tau phosphorylation, and the mitigation of apoptosis.

## 2. Results

### 2.1. Effects of EC on Daily Behavior and Memory in APP/PS1 Transgenic Mice

**Figure 1:**
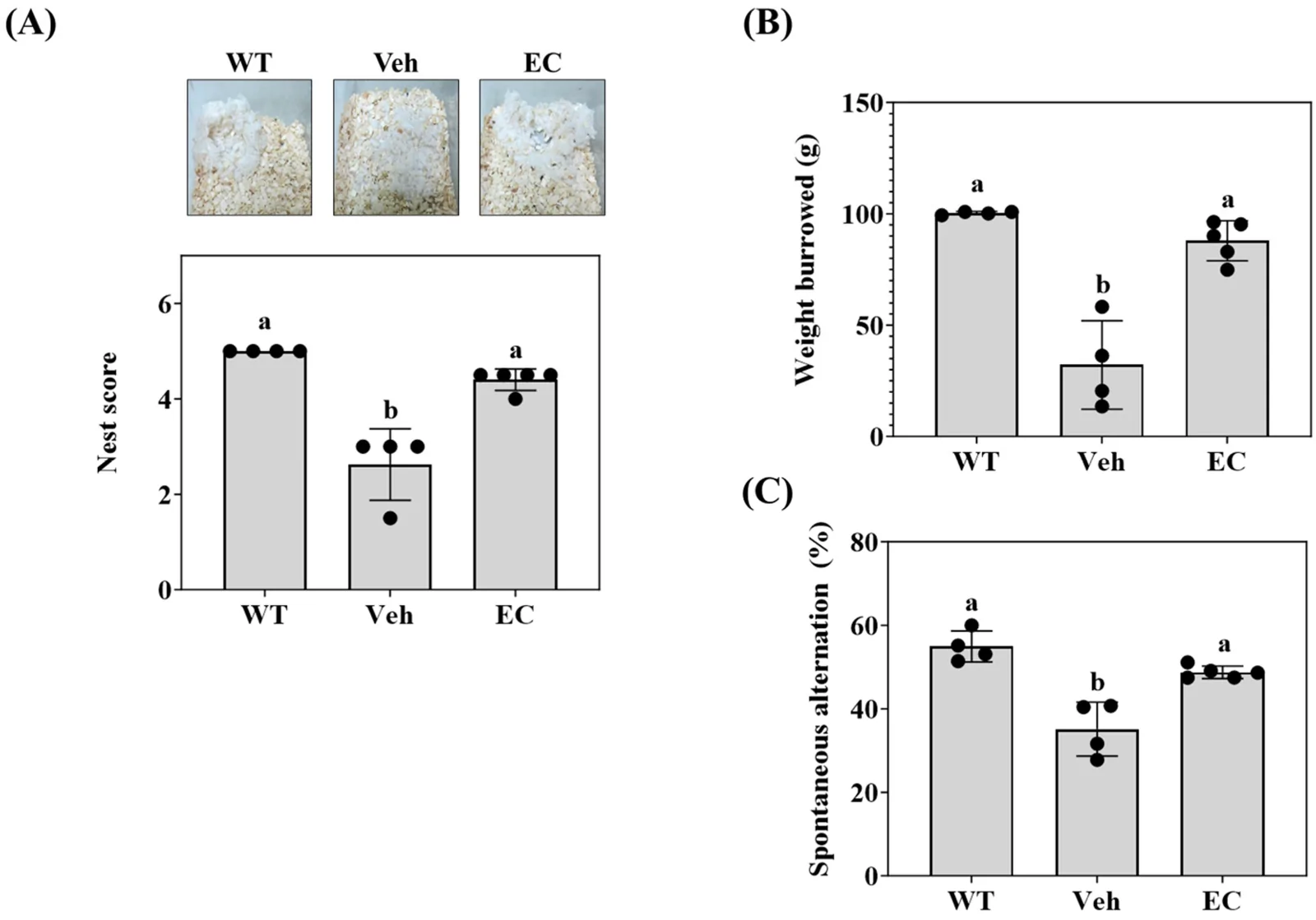
Effects of erinacine C (EC) on behavioral deficits and spatial memory in APP/PS1 transgenic mice. APP/PS1 transgenic mice were orally administered EC at 100 mg/kg/day for 16 weeks (EC group; n = 5). (**A**) Nest-building scores assessed by the nesting task, with representative images of nests shown above the bars. (**B**) Burrowing activity quantified by the weight of materials burrowed. (**C**) Assessment of spatial memory retention via the Y-maze test, expressed as the percentage of spontaneous alternation. APP/PS1 mice treated with the vehicle served as the transgenic control group (Veh group; n = 4), while wild-type C57BL/6J mice served as the non-transgenic control (WT group; n = 4). Data are presented as means ± SD. Bars not sharing a common lowercase letter indicate significant differences (*p* < 0.05) according to one-way ANOVA followed by Tukey’s multiple comparisons test.

Nesting and burrowing are innate behaviors in rodents. In AD experimental models, the assessment of these behaviors is frequently employed to evaluate deficits or improvements in ADL [38,39,40]. As shown in Figure 1A, the nesting scores of the Vehicle (Veh) group were significantly lower than those of the wild-type (WT) group, with a reduction of approximately 48% (*p* < 0.05), indicating abnormal ADL in the transgenic mice. Following EC treatment, nesting scores significantly increased by approximately 68% compared to the Veh group (*p* < 0.05). Similarly, burrowing activity (Figure 1B) was significantly reduced in the Veh group by approximately 68% compared to the WT group (*p* < 0.05). EC treatment significantly enhanced burrowing activity by approximately 173% compared to the Veh group (*p* < 0.05). Furthermore, the Y-maze test was used to assess spontaneous alternation, a key indicator of short-term spatial memory. Results (Figure 1C) demonstrated that the spontaneous alternation rate in the Veh group was significantly lower than in the WT group by 36% (*p* < 0.05). Administration of EC significantly increased this rate by 39% compared to the Veh group (*p* < 0.05).

### 2.2. Effects of EC on Systemic and Hippocampal Inflammatory Responses in APP/PS1 Transgenic Mice

**Figure 2:**
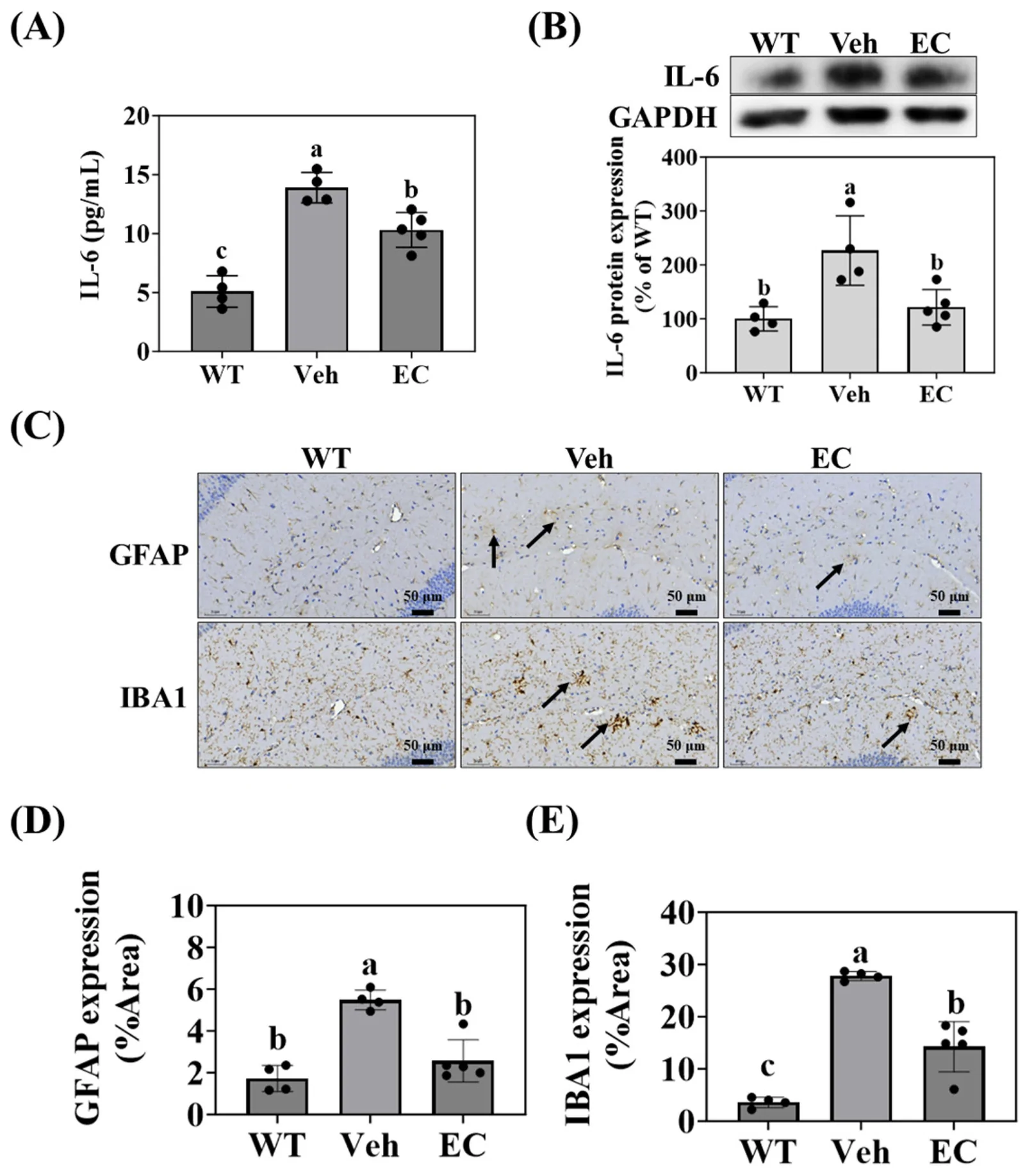
Effects of EC on systemic and hippocampal neuroinflammation in APP/PS1 transgenic mice. APP/PS1 transgenic mice were orally administered EC at 100 mg/kg/day (EC group; n = 5) for 16 weeks. (**A**) Serum levels of interleukin (IL)-6 as measured by ELISA. (**B**) Representative Western blot bands and quantitative analysis of IL-6 protein expression in the hippocampus, with GAPDH used as an internal control. (**C**) Representative immunohistochemical (IHC) staining images of glial fibrillary acidic protein (GFAP) for astrocytes and ionized calcium-binding adapter molecule 1 (IBA1) for microglia in the hippocampus. Scale bar = 50 μm (40× magnification) for representative details. Arrows indicate activated glial cells with positive staining. Quantitative analysis of (**D**) GFAP and (**E**) IBA1 positive area (% Area) was conducted across the whole hippocampal region (scale bar = 200 μm). APP/PS1 mice treated with the vehicle served as the transgenic control group (Veh group; n = 4), while wild-type C57BL/6J mice served as the non-transgenic control (WT group; n = 4). For (**A**,**B**,**D**,**E**), data are presented as means ± SD. Bars not sharing a common lowercase letter indicate significant differences (*p* < 0.05) according to one-way ANOVA followed by Tukey’s multiple comparisons test.

Aβ accumulation activates microglia and astrocytes, leading to the secretion of pro-inflammatory cytokines such as IL-6, which causes neuronal damage [7,8]. As shown in Figure 2A, serum IL-6 concentrations in the Veh group increased significantly by approximately 173% compared to the WT group, while EC treatment reduced these levels by 26% (*p* < 0.05). Moreover, IL-6 protein expression in the hippocampus of the Veh group was 126% higher than in the WT group (Figure 2B; *p* < 0.05) and was subsequently reduced by 46% following EC administration (*p* < 0.05). We further investigated glial activation through immunohistochemistry (IHC) staining of glial fibrillary acidic protein (GFAP; for astrocytes) and ionized calcium-binding adapter molecule 1 (IBA1; for microglia). Quantitative analysis of the hippocampal sections (Figure 2C–E) revealed that the immunoreactivity of both GFAP and IBA1 was significantly elevated in the Veh group compared to the WT group (*p* < 0.05). In contrast, EC treatment noticeably attenuated these inflammatory signals, as evidenced by a significant reduction in the positive immunoreactive areas of both GFAP and IBA1 in the hippocampal tissue.

### 2.3. Effects of EC on Hippocampal Aβ, tau, GSK3β, and Cleaved-Caspase-3 Protein Expression in APP/PS1 Transgenic Mice

**Figure 3:**
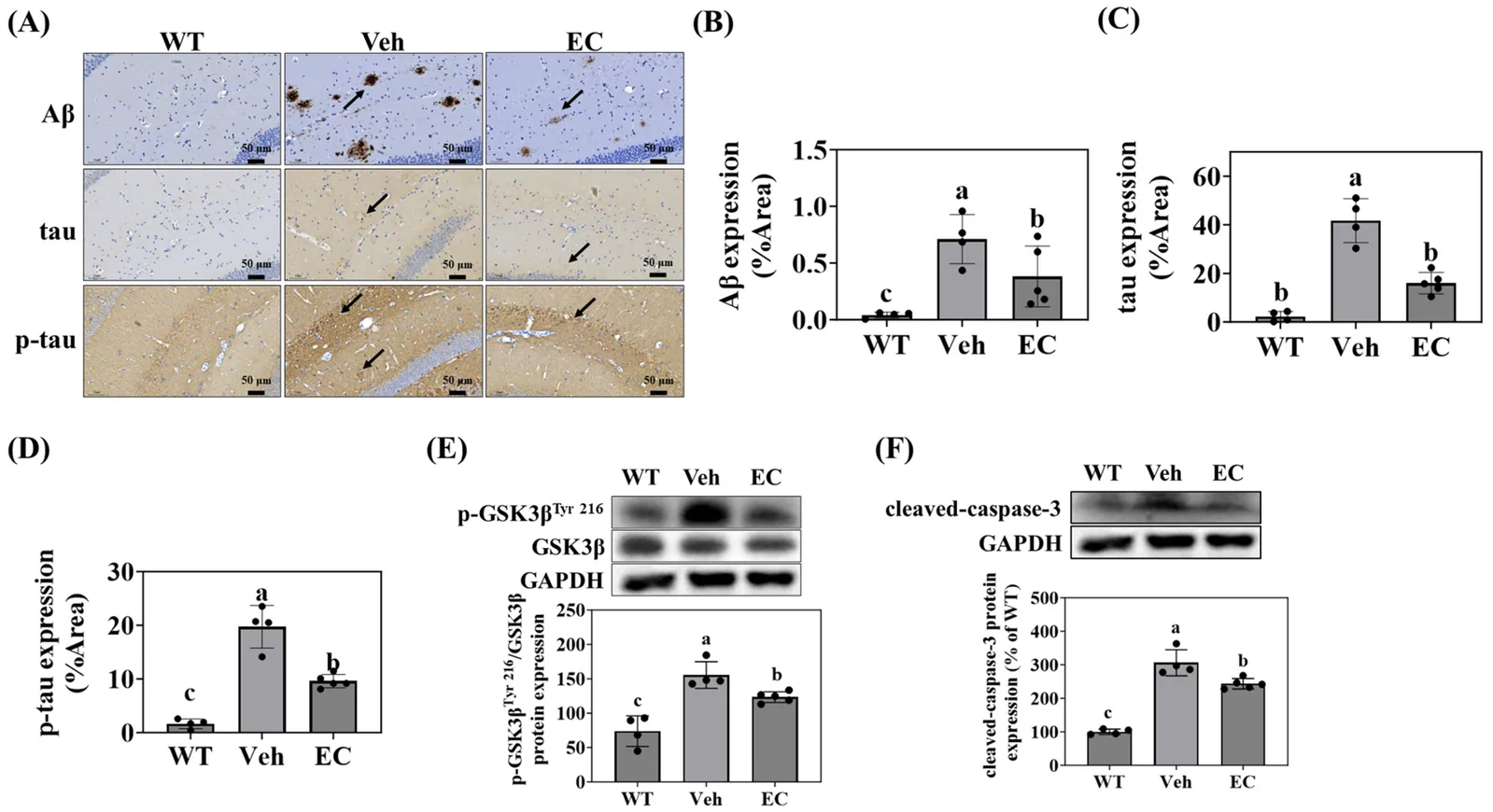
Effects of EC on Aβ accumulation, tau phosphorylation, GSK3β activation, and apoptosis in the hippocampus of APP/PS1 transgenic mice. APP/PS1 transgenic mice were orally administered EC at 100 mg/kg/day (EC group; n = 5) for 16 weeks. (**A**) Representative immunohistochemical (IHC) staining images of Aβ plaques, total tau, and phosphorylated tau (p-tau) in the hippocampus. Scale bar = 50 μm (40× magnification) for representative details. Arrows indicate positive staining or plaque formation. Quantitative analysis of (**B**) Aβ, (**C**) total tau and (**D**) p-tau positive area (% Area) was conducted across the whole hippocampal region (scale bar = 200 μm). (**E**,**F**) Representative Western blot bands and quantitative analysis of (**E**) the ratio of p-GSK3β^Tyr216^ to total GSK3β and (**F**) cleaved-caspase-3 protein levels in the hippocampus. GAPDH was used as the internal control for protein loading. APP/PS1 mice treated with the vehicle served as the transgenic control group (Veh group; n = 4), while wild-type C57BL/6J mice served as the non-transgenic control (WT group; n = 4). For (**B**–**F**), data are presented as means ± SD. Bars not sharing a common lowercase letter indicate significant differences (*p* < 0.05) according to one-way ANOVA followed by Tukey’s multiple comparisons test.

Pathological hallmarks of AD include the accumulation of Aβ plaques and hyperphosphorylated tau proteins [41,42]. As shown in the representative immunohistochemical images in Figure 3A, a substantial presence of Aβ plaques and robust p-tau staining were observed in the hippocampal sections of the Veh group, whereas the EC-treated group displayed a clear reduction in both plaque density and phosphorylation signals. Quantitative analysis of the hippocampal sections further confirmed that the expression levels of Aβ (Figure 3B), tau (Figure 3C), and p-tau (Figure 3D) were significantly elevated in the Veh group compared to the WT group (*p* < 0.05), while EC treatment significantly attenuated these pathological increases (*p* < 0.05). Furthermore, the ratio of p-GSK3β^Tyr216^/total GSK3β in the Veh group was 110% higher than in the WT group (Figure 3E; *p* < 0.05), while EC treatment significantly decreased this ratio by approximately 21% (*p* < 0.05). Additionally, the expression of the apoptotic marker cleaved-caspase-3 was 206% higher in the Veh group compared to the WT group (Figure 3F; *p* < 0.05). EC administration significantly suppressed cleaved-caspase-3 expression by 20% compared to the Veh group (*p* < 0.05).

### 2.4. Effects of EC on Aβ_25-35_-Induced Cytotoxicity in PC12 Cells

**Figure 4:**
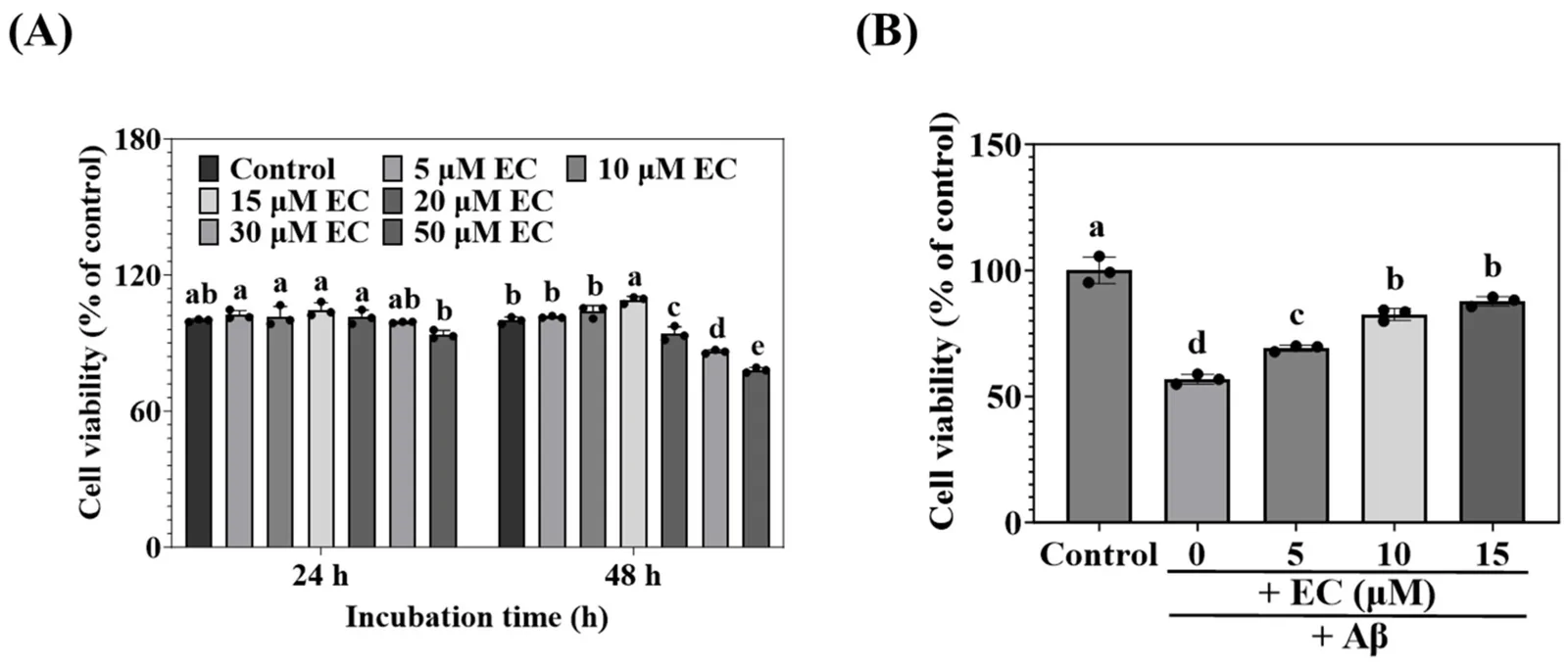
Effects of erinacine C (EC) on PC12 cell viability and its protective effect against Aβ_25-35_-induced cytotoxicity. (**A**) Assessment of EC cytotoxicity. PC12 cells (5 × 10^5^ cells/mL) were treated with various concentrations of EC (5–50 μM) for 24 and 48 h. (**B**) Protective effects of EC against Aβ_25-35_-induced toxicity. PC12 cells were pretreated with EC (5, 10, and 15 μM) for 24 h, followed by exposure to 20 μM Aβ_25-35_ for an additional 24 h. Cell viability was determined using the MTT assay and expressed as a percentage of the control group. Data are presented as means ± SD (n = 3). Bars not sharing a common lowercase letter indicate significant differences (*p* < 0.05) according to one-way ANOVA followed by Tukey’s multiple comparisons test.

To investigate the mechanisms by which EC ameliorates AD symptoms through anti-apoptotic pathways, an in vitro model using rat pheochromocytoma (PC12) cells challenged with Aβ_25-35_ was employed. PC12 cells were treated with EC (5–50 μM) for 24 and 48 h. As shown in Figure 4A, within the concentration range of 5 to 15 μM, EC exhibited high safety and was well tolerated by PC12 cells at both 24 and 48 h (*p* > 0.05). Although the 48 h assessment confirmed the long-term safety profile of EC, a 24 h pretreatment window was selected for the subsequent neuroprotective and signaling evaluations. This was designed to optimally capture acute protective responses against Aβ_25-35_-induced toxicity while preventing potential experimental variables associated with prolonged in vitro culture. However, a decrease in cell viability was observed at 50 μM at 24 h, and at 20, 30, and 50 μM at 48 h. Thus, 5, 10, and 15 μM were selected for subsequent experiments. Exposure to 20 μM Aβ_25-35_ for 24 h significantly induced cell death by 43% compared to the control (Figure 4B; *p* < 0.05). Pretreatment with 5, 10, and 15 μM EC significantly improved cell viability by 12%, 26%, and 31%, respectively (*p* < 0.05).

### 2.5. Effects of EC on Cell Cycle and Apoptosis in Aβ_25-35_-Induced PC12 Cells

**Figure 5:**
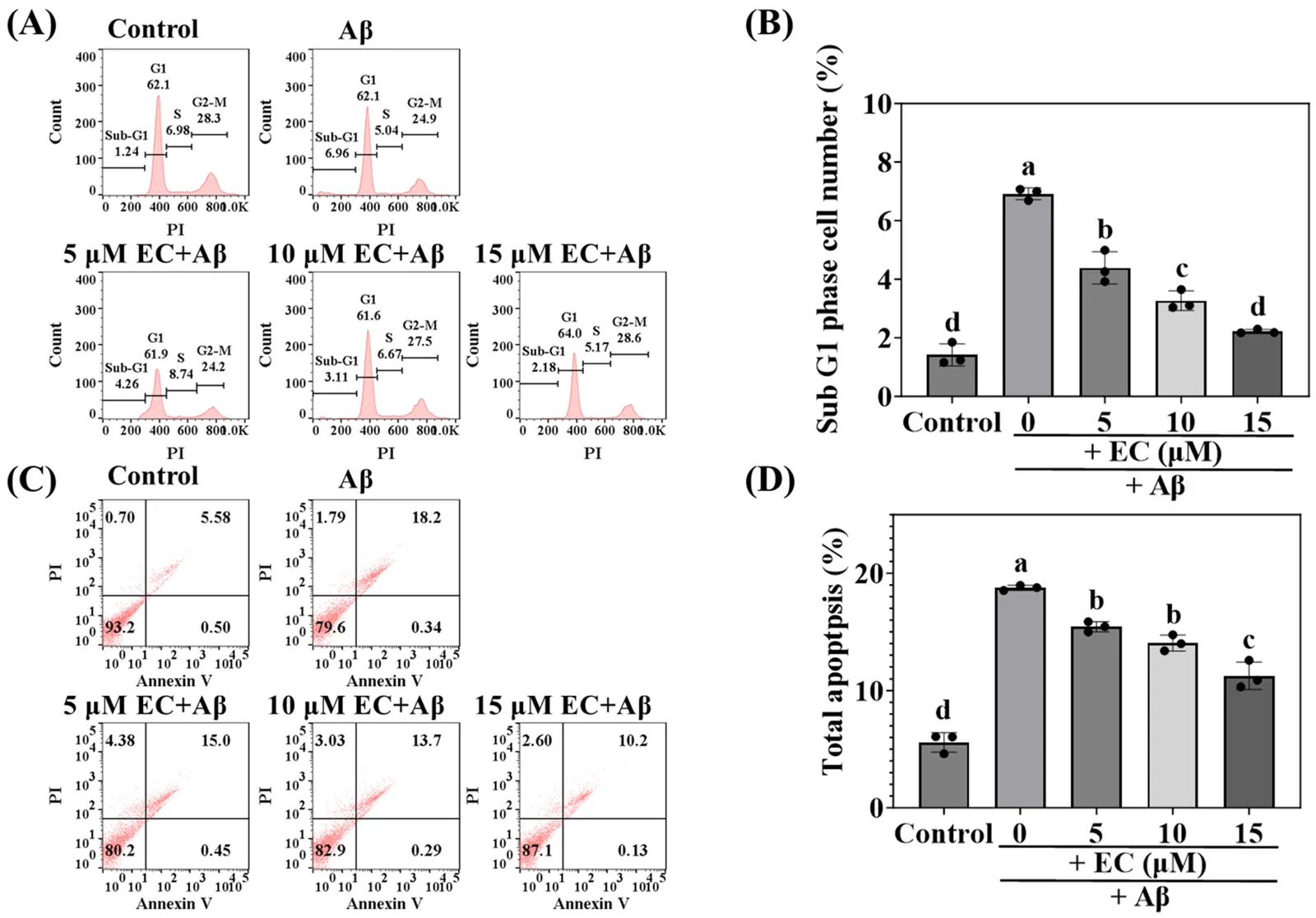
Effects of erinacine C (EC) on Aβ_25-35_-induced cell cycle redistribution and apoptosis in PC12 cells. PC12 cells (5 × 10^5^ cells/mL) were pretreated with EC (5, 10 and 15 μM) for 24 h, followed by exposure to 20 μM Aβ_25-35_ for an additional 24 h. (**A**) Representative flow cytometry histograms showing cell cycle distribution. (**B**) Quantitative analysis of the percentage of cells in the sub-G1 phase. (**C**) Representative flow cytometry scatter plots of Annexin V-FITC/PI staining for apoptosis detection. (**D**) Quantification of the total apoptosis rate, representing the sum of early and late apoptotic cells. For (**B**,**C**), data are presented as means ± SD (n = 3). Bars not sharing a common lowercase letter indicate significant differences (*p* < 0.05) according to one-way ANOVA followed by Tukey’s multiple comparisons test.

Flow cytometry was used to determine whether the protective effect of EC was associated with the inhibition of apoptosis. As shown in Figure 5B, treatment with 20 μM Aβ_25-35_ increased the sub-G1 population by 388% compared to the control (*p* < 0.05). Pretreatment with 5, 10, and 15 μM EC reduced the sub-G1 population by 37%, 53%, and 68%, respectively, compared to the Aβ_25-35_ group (*p* < 0.05). Annexin V/PI dual staining revealed that 20 μM Aβ_25-35_ increased the total apoptosis rate by 236% compared to the control (Figure 5D; *p* < 0.05). Treatment with 5, 10, and 15 μM EC significantly decreased total apoptosis by 18%, 25%, and 40%, respectively (*p* < 0.05).

### 2.6. Effects of EC on Bcl-2, Bax, and Cleaved-Caspase-3 Expression in Aβ_25-35_-Induced PC12 Cells

**Figure 6:**
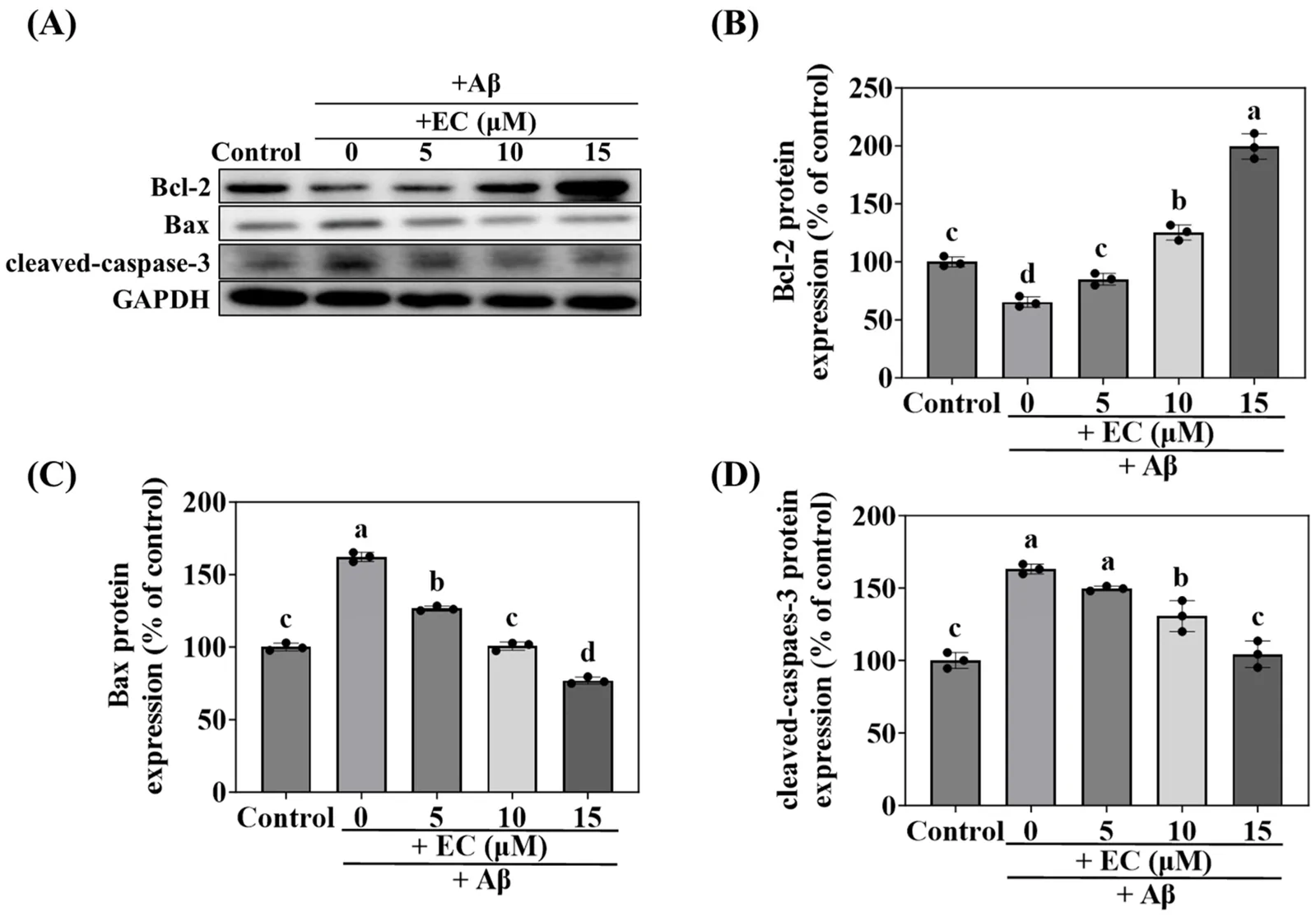
Effects of erinacine C (EC) on Aβ_25-35_-induced apoptosis-related protein expression in PC12 cells. PC12 cells (5 × 10^5^ cells/mL) were pretreated with EC (5, 10 and 15 μM) for 24 h, followed by exposure to 20 μM Aβ_25-35_ for an additional 24 h. (**A**) Representative Western blot bands showing the protein levels of the anti-apoptotic protein Bcl-2, the pro-apoptotic protein Bax, and the executioner caspase, cleaved-caspase-3. GAPDH was used as the internal loading control. (**B**–**D**) Quantitative densitometric analysis of (**B**) Bcl-2, (**C**) Bax, and (**D**) cleaved-caspase-3 protein expression. For (**B**–**D**), data are normalized to the control group and presented as means ± SD (n = 3). Bars not sharing a common lowercase letter indicate significant differences (*p* < 0.05) according to one-way ANOVA followed by Tukey’s multiple comparisons test.

The molecular mechanisms of apoptosis were further elucidated. As shown in Figure 6, 20 μM Aβ_25-35_ reduced Bcl-2 expression by 35% compared to the control. Pretreatment with 5, 10, and 15 μM EC significantly increased Bcl-2 expression by 30%, 92%, and 205% compared to the Aβ_25-35_ group (Figure 6B; *p* < 0.05). Conversely, Aβ_25-35_ increased Bax expression by 62%, whereas EC at 5, 10, and 15 μM reduced it by 22%, 38% and 53%, respectively (Figure 6C; *p* < 0.05). Furthermore, Aβ_25-35_ increased cleaved-caspase-3 levels by 63% (*p* < 0.05), while EC treatment at 10 and 15 μM suppressed its expression by 20%, and 36%, respectively (Figure 6C; *p* < 0.05).

### 2.7. Effects of EC on tau, GSK3β, and Akt Expression in Aβ_25-35_-Induced PC12 Cells

**Figure 7:**
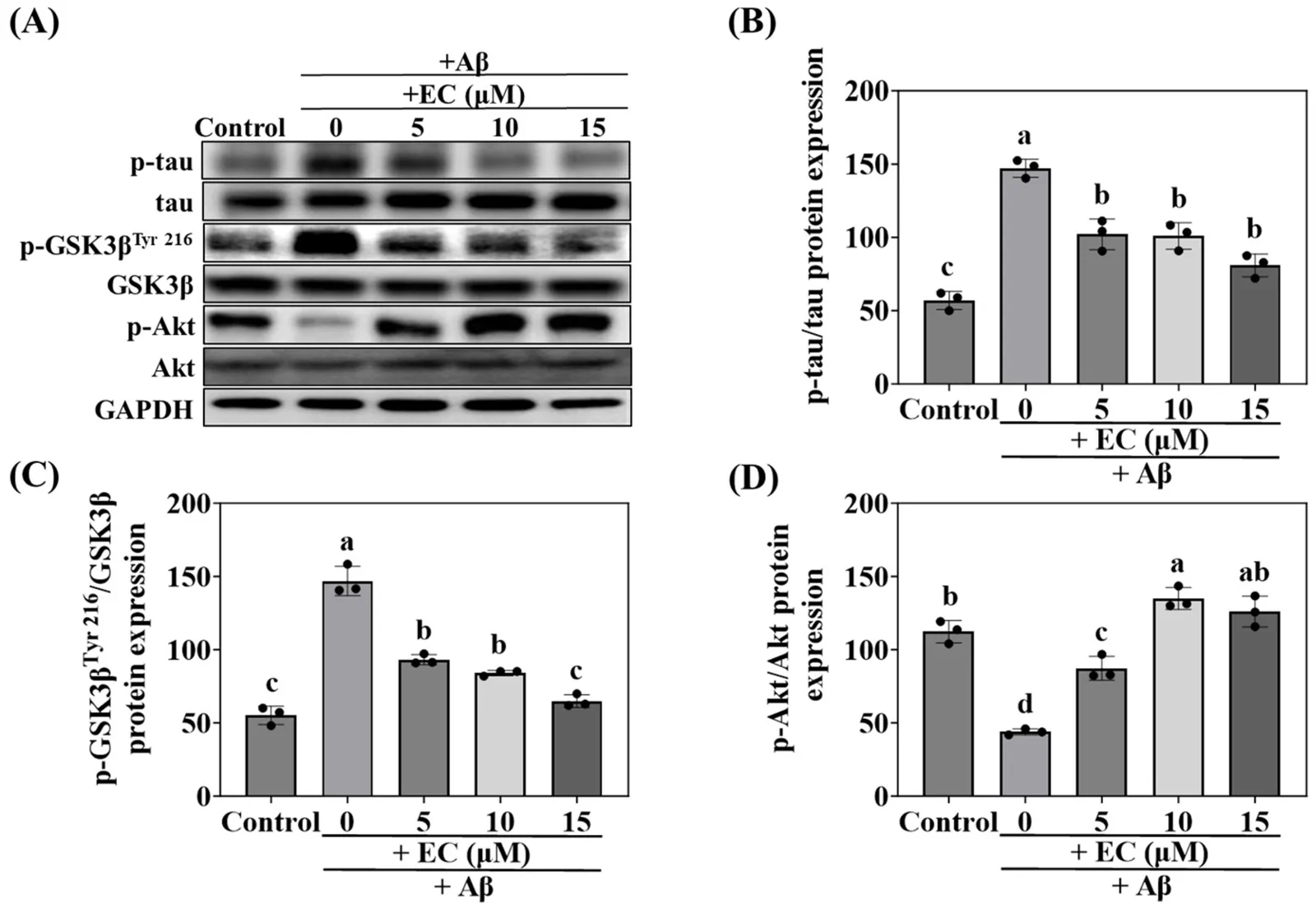
Effects of erinacine C (EC) on Aβ_25-35_-induced phosphorylation of tau, GSK3β, and Akt in PC12 cells. PC12 cells (5 × 10^5^ cells/mL) were pretreated with EC (5, 10 and 15 μM) for 24 h, followed by exposure to 20 μM Aβ_25-35_ for an additional 24 h. (**A**) Representative Western blot bands showing the protein levels of phosphorylated tau (p-tau), total tau, p-GSK3β^Tyr216^, total GSK3β, p-Akt, and total Akt. GAPDH was used as the internal loading control. (**B**–**D**) Quantitative densitometric analysis of the ratios of (**B**) p-tau/total tau, (**C**) p-GSK3β^Tyr216^/total GSK3β, and (**D**) p-Akt/total Akt. For (**B**–**D**), data are presented as means ± SD (n = 3). Bars not sharing a common lowercase letter indicate significant differences (*p* < 0.05) according to one-way ANOVA followed by Tukey’s multiple comparisons test.

Mechanism analysis (Figure 7A) showed that 20 μM Aβ_25-35_ increased the p-tau/total tau ratio by 158% compared to the control (*p* < 0.05). Pretreatment with 5, 10, and 15 μM EC significantly reduced this ratio by 31%, 31%, and 45%, respectively (Figure 7B; *p* < 0.05). Additionally, Aβ_25-35_ increased the p-GSK3β^Tyr216^/total GSK3β ratio by 166%, which was significantly attenuated by 5, 10, and 15 μM EC by 37%, 43%, and 56%, respectively (Figure 7C; *p* < 0.05). In contrast, Aβ_25-35_ decreased the p-Akt/total Akt ratio by 61%, while treatment with 5, 10, and 15 μM EC significantly elevated this ratio by 99%, 208%, and 187%, respectively (Figure 7D; *p* < 0.05).

## 3. Discussion

This study demonstrates that EC exerts significant neuroprotective effects in both APP/PS1 transgenic mice, which exhibit typical AD pathologies, and Aβ_25-35_-induced PC12 cells.

Our findings reveal that EC not only ameliorates daily behavioral deficits and memory loss in AD mice but also is associated with the attenuation of neuroinflammation and apoptotic cascades alongside changes in the Akt/GSK3β signaling profile. Notably, the reduced Aβ plaque burden in the hippocampus further supports the potential of EC to attenuate AD-like pathological features in vivo.

Although clinical trials have indicated that daily dietary intake of *Hericium erinaceus* whole fruiting body powder can temporarily improve cognitive scores in individuals with mild cognitive impairment, these beneficial effects decline upon cessation of supplementation [43]. This limitation is suggested to be rooted in the chemical and pharmacological constraints of crude mushroom matrices. First, secondary metabolite concentrations within whole mushrooms fluctuate depending on cultivation substrates and growth environments, limiting experimental and therapeutic reproducibility [30]. Second, the culinary use of whole mushrooms primarily delivers fruiting body-derived hericenones; although these compounds demonstrate neuroprotective and NGF-stimulatory properties in cellular models [44], their efficiency in crossing the blood–brain barrier (BBB) remains a subject of ongoing investigation compared to mycelia-derived diterpenoids. Notably, research indicates that neuroprotective erinacines are predominantly identified in the mycelia and remain scarce or largely undetected in the fruiting bodies [32,36,37,45]. In contrast, our study minimizes these matrix-related confounding factors by strategically isolating pure EC from the mycelia.

This targeted approach guarantees a standardized dosage free from whole-food molecular interference and leverages the compound’s intrinsic physicochemical properties to achieve consistent neuroprotective outcomes, as validated by the behavioral and molecular restorations observed herein. While direct pharmacokinetic and brain tissue distribution studies of EC remain to be conducted in future work, its low molecular weight and lipophilicity may provide a rationale for its observed central efficacy.

APP/PS1 mice are a robust animal model for AD research, simulating the excessive accumulation of amyloid plaques and subsequent neurodegenerative changes observed in the human brain [46,47,48]. Nesting and burrowing are essential innate behaviors in rodents and are considered critical indicators for assessing ADL in AD models [37,38,39]. In the present study, APP/PS1 mice showed a significant decline in nesting and burrowing capabilities compared to wild-type mice, aligning with the previous literature which indicates that Aβ deposition impairs frontal cortex and hippocampal functions [38,39,40,49]. Following EC treatment, these instinctive behaviors were substantially restored; notably, burrowing activity was increased nearly three-fold. Furthermore, EC treatment markedly enhanced the spontaneous alternation rate in the Y-maze test, suggesting that EC possesses the potential to alleviate AD-related cognitive decline and behavioral dysfunction, effectively maintaining the functional integrity of the nervous system.

During the pathological progression of AD, excessive Aβ deposition triggers microglia and astrocytes to secrete a battery of pro-inflammatory cytokines, leading to chronic neuronal damage [7,8,9,10]. We observed a significant up-regulation of GFAP and IBA1 expression in the hippocampus of APP/PS1 mice, accompanied by elevated systemic IL-6 levels. These results suggest that over-activation of glial cells contributes to a potent inflammatory milieu. EC treatment effectively reduced IL-6 concentrations in both serum and hippocampal tissues while suppressing the expression of GFAP and IBA1.

Although direct Aβ production or enzymatic clearance pathways were not explicitly quantified in this study, the observed reduction in overall Aβ plaque burden indicates a favorable impact on plaque accumulation.

These findings are consistent with our previous study, which demonstrated that EC reduced the secretion of IL-6 in LPS-induced BV2 microglia [50]. Furthermore, EC not only inhibits NF-κB signaling but also strengthens cellular defense mechanisms by activating the Nrf2/HO-1 antioxidant pathway [50].

Taken together, these results suggest that EC may mitigate Aβ-induced inflammatory damage associated with dampened glial activation and the subsequent release of inflammatory mediators.

The abnormal activation of GSK3β is a primary driver of tau hyperphosphorylation in AD pathology [51]. Akt serves as an upstream survival factor that is widely recognized to regulate GSK3β; conversely, Aβ stress diminishes Akt activity, leading to altered GSK3β regulation and phosphorylation at its activation site (Tyr216) [19,52]. Our data confirm that Aβ_25-35_ induction led to a 61% decrease in the p-Akt/Akt ratio and a 166% increase in the p-GSK3β^Tyr216^/GSK3β ratio in PC12 cells. EC intervention significantly reversed this imbalance, restoring p-Akt levels by approximately 1.8- to 2-fold and suppressing the aberrant activation of GSK3β. Consequently, a significant reduction in the p-tau/tau ratio (31% to 45%) was observed in both hippocampal tissues and PC12 cells. While the current study highlights the concurrent modulation of p-Akt and p-GSK3β (Tyr216), it should be noted that other parallel signal mediators or upstream regulators may also participate in this network.

We further explored the anti-apoptotic mechanisms of EC using the Aβ_25-35_-induced PC12 cell model. Flow cytometric analysis showed that EC significantly lowered the sub-G1 population and the total apoptosis rate. At the molecular level, EC treatment was accompanied by the up-regulation of the anti-apoptotic protein Bcl-2 and the down-regulation of the pro-apoptotic protein Bax and cleaved-caspase-3. This indicates that beyond reducing overall Aβ plaque accumulation, EC is associated with the attenuation of the Aβ_25-35_-induced apoptotic cascade to assist in maintaining neuronal integrity.

However, certain limitations remain regarding the mechanistic depth of Akt/GSK3β regulation. Since genetic silencing or specific inhibitors were not employed in this study for loss-of-function validation, a direct causal relationship for this pathway cannot be definitively concluded, and the involvement of parallel regulatory mediators cannot be entirely excluded. Additionally, direct pharmacokinetics and Aβ metabolic turnover mechanisms warrant further verification. Future research will focus on utilizing knockout models, specific kinase inhibitors, and pharmacokinetic tracking to confirm these precise mechanisms. Concurrently, EC represents a promising therapeutic candidate for the prevention and intervention of Alzheimer’s disease.

## 4. Materials and Methods

### 4.1. Chemicals and Reagents

The EC (purity > 98%, molecular formula C_25_H_38_O_6_, molecular weight 434) was a gift from Dr. Chien-Chih Chen (Chang Gung University of Science and Technology, Taoyuan city, Taiwan). Aβ_25-35_ (Cat. No. A4559) and propidium iodide (PI; Cat. No. P4170) were purchased from Sigma-Aldrich (St. Louis, MO, USA). The Annexin V-FITC/PI apoptosis detection kit was purchased from BD Biosciences (Cat. No. 556547; San Jose, CA, USA). RPMI 1640 medium, horse serum (HS), fetal bovine serum (FBS), L-glutamine, sodium bicarbonate and anti-Bax (Cat. No. MA5-14003), anti-caspase 3 (Cat. No. PA5-114687), GFAP (Cat. No. PA5-16291), anti-IBA1 (Cat. No. PA5-27436), anti-tau (Cat. No. 13-6400), anti-p-tau (Cat. No. 35-5300), anti-GSK3β (Cat. No. MA5-15597) were purchased from Invitrogen (Thermo Fisher Scientific, Waltham, MA, USA). The mouse IL-6 ELISA kit was purchased from BioLegend, Inc. (Cat. No. 431315; San Diego, CA, USA). Anti-Bcl-2 (Cat. No. sc-7382), anti-Akt (Cat. No. sc-8312), anti-p-Akt (Cat. No. sc-101629), anti-GAPDH (Cat. No. sc-32233), and anti-p-GSK3β (Cat. No. sc-81496) were purchased from Santa Cruz Biotechnology Inc. (Dallas, TX, USA). Anti-Aβ (Cat. No. GTX134510) was purchased from GeneTex (Irvine, CA, USA).

### 4.2. Animals and Experimental Design

The experimental protocol was approved by the Institutional Animal Care and Use Committee (IACUC) of Hungkuang University (Approval No. HK-10912; approval date: 1 December 2020). Eight-week-old female C57BL/6J mice and ten-week-old female APP/PS1 transgenic mice were purchased from the National Center for Biomodels (NCB, Taipei, Taiwan) and originally obtained from the Jackson Laboratory (No. 034832; Bar Harbor, ME, USA) via Taihe Instruments Co., Ltd. (Taipei, Taiwan), respectively. All mice were housed in a temperature-controlled environment at 22 ± 2 °C with a 12 h light/dark cycle (lights on from 08:00 to 20:00). After a period of acclimation and growth, the mice were assigned to three experimental groups: (1) WT group (WT, n = 4): Normal female C57BL/6J mice (14 weeks old at the beginning of the experiment, mean body weight 21 ± 1.2 g) receiving 0.5% carboxymethyl cellulose (CMC) vehicle. (2) Veh group (Veh, n = 4): Female APP/PS1 mice (16 weeks old at the beginning of the experiment, mean body weight 20.5 ± 1.2 g) receiving 0.5% CMC vehicle. (3) EC group (EC, n = 5): Female APP/PS1 mice (16 weeks old at the beginning of the experiment, mean body weight 20.1 ± 0.9 g) receiving EC at a dosage of 100 mg/kg/day. EC was suspended in 0.5% CMC and administered daily via oral gavage for 16 consecutive weeks, following general animal experimental protocols described previously [52,53] with minor modifications for this transgenic mouse model. The WT and Veh groups were administered an equivalent volume of 0.5% CMC. Throughout the experimental period, body weight, food intake, and water consumption were monitored weekly. In the 15th week, behavioral assessments—including the nesting test, burrowing test, and Y-maze test—were conducted, followed by the collection of blood for serum IL-6 analysis. At the end of the 17th week, all mice were anesthetized with isoflurane and sacrificed. Brain tissues were then harvested for subsequent biochemical analyses and immunohistochemical staining.

### 4.3. Nesting Test

To evaluate the ADL and innate motivated behavior, a nesting test was performed in the 15th week of the experiment following the protocol established by Deacon [53]. Mice were individually housed in separate cages and provided with a 3 × 3 square cotton pieces (Nestlet) approximately one hour before the onset of the dark cycle. The cages were left undisturbed overnight, and nesting performance was assessed the following morning. The quality of the nest was evaluated using a 5-point scoring scale based on the degree of Nestlet shredding and the resulting nest morphology: Score 1: The Nestlet was virtually untouched (<10% shredded); Score 2: The Nestlet was partially shredded (10% to 50% shredded); Score 3: The Nestlet was mostly shredded (50% to 90% shredded), with pieces scattered across the cage without forming a distinct nest; Score 4: The Nestlet was substantially shredded (>90% shredded), with pieces gathered into a flat heap; Score 5: The Nestlet was completely shredded (>90% shredded), and formed into a functional, crater-shaped nest with high walls surrounding the mouse.

### 4.4. Burrowing Test

The burrowing test was performed in the 15th week of the experiment, following the methodology described by Deacon [54]. The burrowing apparatus consisted of a PVC pipe (20 cm in length, 6.8 cm in diameter) with one end sealed and the other end open. Two 5 cm screws were fixed 1 cm from the open end, spaced 3 cm apart, to serve as a supportive base for the tube. Two hours before the onset of the dark cycle, each tube was filled with 230 g of standard laboratory diet and placed into the individual home cage of each mouse. After two hours, the amount of diet displaced from the tube (burrowed out) was collected and weighed. Burrowing activity was quantified as the total weight of the food pellets removed from the tube during the test period.

### 4.5. Y-Maze Spontaneous Alternation Test

Spatial working memory was assessed using the Y-maze spontaneous alternation test, following the method described by Kouémou et al. [55] and Miedel et al. [56]. The Y-maze apparatus consisted of three identical arms (35 cm long, 8 cm wide, and 15 cm high) labeled A, B, and C. Each mouse was placed at the end of one arm and allowed to explore the maze freely for 8 min. The sequence and total number of arm entries were recorded. An alternation was defined as consecutive entries into all three different arms (e.g., ABC, BCA, CAB, BAC or ACB). The percentage of spontaneous alternation was calculated using the following formula: Spontaneous Alternation (%) = Number of Alternations/(Total Arm Entries − 2) × 100.

### 4.6. Serum Inflammatory Cytokine Analysis

Serum IL-6 concentrations were measured using a commercial ELISA kit according to the manufacturer’s instructions. Briefly, samples and standards were incubated in pre-coated 96-well plates for 2.5 h, followed by sequential incubation with a biotinylated detection antibody (1 h) and HRP–streptavidin (45 min). Between each step, the plates were washed four times with 1X wash buffer. The chromogenic reaction was initiated by adding TMB substrate for 30 min in the dark and terminated with a stop solution. The absorbance was measured at 450 nm using a microplate reader. The final IL-6 concentrations were calculated based on a linear standard curve after background correction.

### 4.7. Immunohistochemical Staining

Immunohistochemical staining was performed using the IHC Select^®^ HRP-DAB Kit (Chemicon International Inc., Temecula, CA, USA) to evaluate the protein expression of Aβ, tau, p-tau, GFAP, and IBA1 in brain tissues [57].

Paraffin-embedded sections mounted on glass slides were first incubated in a 70 °C water bath for 30 min.

The sections were then deparaffinized with xylene and rehydrated through a graded series of ethanol. To block endogenous peroxidase activity, the slides were treated with 3% H_2_O_2_ for 2 min. After incubation with a blocking agent to minimize non-specific binding, the sections were incubated with specific primary antibodies against the target proteins. Subsequently, the slides were sequentially incubated with the secondary antibody and streptavidin–HRP. The immunoreactivity was visualized using 3,3′-diaminobenzidine (DAB). The sections were then counterstained with hematoxylin, dehydrated through an ascending ethanol series (75%, 90%, and 100%) and xylene, and finally mounted with mounting medium prior to digital slide scanning.

### 4.8. Image Acquisition and Quantitative Analysis:

Whole-slide digital images of the immunostained brain sections were acquired using a high-resolution digital slide scanner (MoticEasyScan, Motic, Xiamen, China). To quantitatively evaluate target protein expression, the entire hippocampus was quantified for each animal target protein. Quantitative analysis of the positive staining area (% area) was performed using Fiji software (an expanded distribution of ImageJ, version 2.14.0; NIH, Bethesda, MD, USA) on images scanned at 40×; scale bar = 200 μm. For each section, the entire hippocampal region was delineated as a single region of interest (ROI) without subfield division. The percentage of positive area (% Area) for each target protein was calculated following the semi-quantitative IHC analysis protocol described by Crowe & Yue [58]. The intensity threshold for DAB-positive signals was uniformly established across all experimental groups, and target protein expression was expressed as the percentage of DAB-positive area relative to the total area of the delineated hippocampus (% Area).

### 4.9. Cell Culture and Treatments

PC12 cells were purchased from the Biological Resources Conservation and Research Center of the Institute of Food Industry Development (Hsinchu, Taiwan). PC12 cells were cultured in RPMI 1640 medium containing 2 mM L-glutamine, 1.5 g/L sodium bicarbonate, 10% heat-inactivated horse serum, and 5% FBS and 1% penicillin–streptomycin in a humidified incubator at 37 °C with 5% CO_2_. For the cytotoxicity assessment of EC, PC12 cells were seeded at a density of 5 × 10^5^ cells/mL in 24-well plates and treated with various concentrations of EC (5, 10, 15, 20, 30, and 50 μM) for 24 and 48 h. For the neuroprotection study against Aβ_25-35_-induced toxicity, cells were pre-incubated with EC (5, 10, and 15 μM) for 24 h. Subsequently, the cells were exposed to 20 μM Aβ_25-35_ and incubated for an additional 24 h.

### 4.10. Cell Viability Assay

Cell viability was determined using the 3-(4,5-dimethylthiazol-2-yl)-2,5-diphenyltetrazolium bromide (MTT) assay. Briefly, after the designated treatment periods, MTT solution (final concentration: 0.5 mg/mL) was added to each well and the plates were incubated at 37 °C for 1 h to allow the formation of formazan crystals. The culture medium was then removed, and the resulting formazan was dissolved in dimethyl sulfoxide (DMSO). The absorbance was measured at 570 nm using a microplate reader. Cell viability was expressed as a percentage of the control group (untreated cells), which was defined as 100%.

### 4.11. Cell Cycle Analysis

Cell cycle distribution was determined according to the method described by Wang et al. [59]. Following treatment, the culture supernatant was collected, and the cells were washed twice with 2 mL of phosphate-buffered saline (PBS). The cells were detached using trypsin and transferred to centrifuge tubes. After centrifugation at 300× *g* for 5 min, the supernatant was discarded, and the cell pellet was washed twice with 5 mL of PBS to ensure thorough removal of residual medium. For cell fixation, the cell pellet was gently resuspended while slowly adding 5 mL of ice-cold 70% ethanol (pre-cooled to −20 °C). The samples were then stored at −20 °C for 24 h to ensure complete fixation. After fixation, the cells were centrifuged, washed with 1 mL of PBS, and resuspended in 1 mL of staining solution containing 4 μg/mL propidium iodide (PI) and 0.5 mg/mL RNase A. The mixture was incubated in a 37 °C water bath for 30 min in the dark. Finally, the cell cycle distribution was analyzed using a flow cytometer (BD FACSCanto™ II flow cytometer, BD Biosciences, San Jose, CA, USA), with 10,000 events collected per sample. The data were further processed and quantified using FlowJo software (version 10.10.1; Tree Star, Ashland, OR, USA).

### 4.12. Apoptosis Analysis

Cell apoptosis was detected using the Annexin V-FITC and PI double staining method, following the protocol described by Wang et al. [59]. After treatment, both the culture supernatant and the adherent cells were collected and centrifuged at 300× *g* for 5 min. The cell pellets were washed twice with 5 mL of PBS and then resuspended in 100 μL of Annexin V Binding Buffer. Subsequently, 5 μL of Annexin V-FITC and 5 μL of PI were added to the cell suspension. The mixture was incubated in the dark at 37 °C for 30 min. Following incubation, 400 μL of Annexin V Binding Buffer was added to each sample. The apoptotic cells were analyzed using a flow cytometer (BD FACSCanto™ II flow cytometer, BD Biosciences, San Jose, CA, USA), with 10,000 events collected per sample. The data were further processed and quantified using FlowJo software (version 10.10.1; Tree Star, Ashland, OR, USA).

### 4.13. Western Blot Analysis

Protein expression levels of Bcl-2, Bax, GSK-3β, p-GSK3β^Tyr216^, tau, p-tau, Akt, p-Akt, and IL-6 were analyzed via Western blot [60]. Protein samples (30 μg) were mixed with 5× loading dye and denatured at 95 °C for 5 min using a dry bath incubator. After cooling, the samples were loaded into the wells of 10% SDS-PAGE gels and separated by electrophoresis at 80 V and 110 V. For protein transfer, PVDF membranes were activated in methanol and equilibrated in transfer buffer. The proteins were transferred from the gel to the membrane using a sandwich assembly (sponge/filter paper/gel/membrane) at 300 mA for 1.5 h at 4 °C.

Following transfer, the membranes were blocked with 5% non-fat milk at room temperature for 1 h, followed by overnight incubation at 4 °C with specific primary antibodies: anti-Bcl-2 (1:500), anti-Bax (1:1000), anti-Akt (1:500), anti-p-Akt (1:500), anti-Tau (1:1000), anti-p-Tau (1:1000), anti-GSK3β (1:1000), anti-p-GSK3β^Tyr216^ (1:500), and anti-GAPDH (1:500) as an internal loading control. The following day, the primary antibodies were recovered, and the membranes were washed three times with TBST buffer. Subsequently, the membranes were incubated with secondary antibodies for 2 h at room temperature and washed again three times with TBST buffer. Protein bands were visualized using an ECL kit (reagent:oxidizer = 1:1) and captured using a chemiluminescence imaging system (LAS-4000; Fujifilm, Tokyo, Japan). The target protein bands were quantified using AlphaEaseFC (version 4.0.0; Alpha Innotech, San Leandro, CA, USA) software with all target signals normalized to GAPDH.

### 4.14. Statistical Analysis

Statistical analysis was performed as described previously [61,62]. Values were expressed as means ± standard deviation (SD) and analyzed using one-way ANOVA, followed by Tukey’s multiple comparisons test for comparisons among group means. For the animal experiments, the exact number of animals used for each group was as follows: wild-type (WT) group (n = 4), vehicle-treated (Veh) group (n = 4), and erinacine C (EC)-treated group (n = 5). For the in vitro cell experiments, all data were obtained from at least three independent experiments performed in triplicate. The statistical analysis was performed using SPSS version 18 (IBM Inc., Armonk, NY, USA). *p* values < 0.05 were considered statistically significant.

## 5. Conclusions

In conclusion, this study demonstrates that erinacine C (EC)—a purified active component from *Hericium erinaceus* mycelia—effectively ameliorates cognitive decline and behavioral deficits in APP/PS1 mice while counteracting Aβ_25-35_-induced cytotoxicity in PC12 cells. Functional improvements observed with EC intervention are accompanied by reduced neuroinflammation (dampened glial activation and IL-6 levels), lower Aβ plaque burden, and the inhibition of apoptotic markers. Crucially, EC treatment is associated with restored balance in the Akt/GSK3β signaling profile, which correlates with reduced tau hyperphosphorylation and favorable modulation of Bcl-2/Bax/cleaved-caspase-3 signaling.

## Data Availability

The original contributions presented in this study are included in the article. Further inquiries can be directed to the corresponding authors.

## Abbreviations

Aβ	Amyloid-beta
EC	Erinacine C
ADL	Activities of Daily Living
GFAP	Glial Fibrillary Acidic Protein
IBA1	Ionized Calcium-Binding Adapter Molecule 1
AD	Alzheimer’s Disease
NFTs	Neurofibrillary Tangles
IL	Interleukin
TNF-α	Tumor Necrosis Factor-α
Akt	Protein Kinase B
GSK3β	Glycogen Synthase Kinase 3 beta
BBB	Blood–Brain Barrier
NGF	Nerve Growth Factor
PC12 cells	Rat Pheochromocytoma PC12 Cells
IHC	Immunohistochemistry
WT	Wild-Type
Nrf2	Nuclear Transcription Factor Erythroid 2-Related Factor
NF-κB	Nuclear Factor Kappa-Light-Chain-Enhancer of Activated B Cells
HO-1	Heme Oxygenase-1
LPS	Lipopolysaccharide
HRP	Horseradish Peroxidase
PBS	Phosphate-Buffered Saline
TMB	3,3′,5,5′-Tetramethylenzidin
DAB	3,3′-Diaminobenzidine
MTT assay	3-(4,5-Dimethylthiazol-2-yl)-2,5-Diphenyltetrazolium Bromide assay
DMSO	Dimethyl Sulfoxide
PI;	Propidium Iodide
PVDF	Polyvinylidene Difluoride
SDS-PAGE	Sodium Dodecyl Sulfate–Polyacrylamide Gel Electrophoresis
TBST	Tris-Buffered Saline with Tween 20
